# Physiological Demands and Muscle Activity of Jockeys in Trial and Race Riding

**DOI:** 10.3390/ani12182351

**Published:** 2022-09-08

**Authors:** Kylie Legg, Darryl Cochrane, Erica Gee, Paul Macdermid, Chris Rogers

**Affiliations:** 1 School of Veterinary Science, Massey University, Private Bag 11-222, Palmerston North 4410, New Zealand; 2 School of Sport, Exercise and Nutrition, Massey University, Private Bag 11-222, Palmerston North 4442, New Zealand; 3 School of Agriculture and Environment, Massey University, Private Bag 11-222, Palmerston North 4442, New Zealand

**Keywords:** Thoroughbred racing, exercise, training, equine, jockey

## Abstract

**Simple Summary:**

Jockeys are elite athletes and their performance during a race impacts not only their own injury risk and career longevity but also that of the horse they ride. The physiological parameters and muscle activity of jockeys during trials and races were quantified. This study found that trials act as a segue to race riding, with jockeys experiencing moderate to high-intensity effort during a trial, but using both their legs and (increasingly) arms to dampen horse oscillation. Jockeys riding in races exercise at near maximal physiological potential, using only their legs to dampen horse oscillation in a lower crouched posture than that adopted by jockeys in trials, with their centre of mass (COM) shifted anteriorly. Therefore, the competition (race) level performance demands of the jockey are not only higher than training level demands, but jockeys assume a different riding posture. Achieving race-specific fitness in readiness for competition is important for both horse and jockey safety, performance, and career longevity. Future physical training guidelines should aim to specifically target the physiological demands of race riding which are not addressed by training rides.

**Abstract:**

Physiological parameters and muscle activity of jockeys may affect their fall and injury risk, performance, and career longevity, as well as the performance and welfare of the horses they ride. Therefore, this study aimed to quantify the physiological demands, body displacement, and electromyographic (EMG) activity of twelve jockeys riding 52 trials and 16 professional races. The jockeys were instrumented with heart rate (HR) monitors, accelerometers, and integrated EMG clothing (recording eight muscle groups: *quadriceps*, *hamstrings*, *gluteal*, *erector spinae*/*lower back*, *abdominal external obliques*, *abdominal*, *trapezial* and *pectoral*) which recorded continuously whilst riding. During race day, jockeys rode an average of 5 ± 4 trials and 4 ± 2 races over 2–2.5 h. The trials represented lower intensity cardiovascular demand (~81% HRmax) and Training Impulse (TRIMP) scores (4.4 ± 1.8) than races at maximal intensity effort (~94% HRmax, 7.2 ± 1.8 TRIMP, *p* < 0.05). Jockey head displacement was similar in trials (5.4 ± 2.1 cm) and races (5.6 ± 2.2 cm, *p* > 0.05), with more vertical (6.7 ± 2.7 cm) and less medio/lateral (2.3 ± 0.7 cm) and fore/aft (3.7 ± 1.6 cm) displacement for jockeys riding in trials than races (5.5 ± 2.3, 2.8 ± 1.0, 5.6 ± 2.5 cm, *p* < 0.05). Jockeys in races adopted a lower crouched posture, with their centre of mass (COM) shifted anteriorly, using greater *hamstring* activation and less upper arm muscle activation than in trials. The differences in riding posture and physiological demands on jockeys riding in a race rather than a trial, highlight the requirement for an off-horse race-specific training programme to improve jockey fitness and performance. Greater jockey stability and coordination will have mutual benefits for both horse welfare and performance.

## 1. Introduction

Jockeys have an integral role in the quality of racing and the welfare of the racehorse, so ensuring they are performing to their best potential is paramount not only for their own performance, injury risk, and career longevity, but will also impact the horses they ride. Securing a ride in a race, and winning, is the highest form of competition available to a jockey, with every race deemed of high importance due to pressure from trainers and owners, in addition to the prospect of individual financial awards and career progression (hopes of securing future rides). During a race, jockeys ride at close to their maximal physiological (aerobic and anaerobic) capacity [1,2,3], supporting themselves in a crouched posture with only their toes or the ball of the foot in the stirrups, on a horse galloping at speeds exceeding 60 km∙h^−1^ [4]. Continuous quasi-isometric muscle activity alterations in the jockey provide both postural support and dampen horse oscillation, with more experienced riders having greater postural control than novices [5,6,7]. Measurement of the muscle activity and physiological parameters for top-level competitive conditions is rare in any form of sport, due to the (perceived) possibility of interference of the additional instruments with the jockey, compromising their riding performance.

The riding schedule of an apprentice jockey in New Zealand follows the race preparation for horses, and generally consists of approximately 2.5 h of riding multiple horses in “track work” rides at 8–9 m∙s^−1^ 5–6 days a week, with a small proportion of fast workouts (gallop at >14 m∙s^−1^) once or twice a week [8,9,10,11,12]. Jockeys participate in jump out and trial sessions, as well as race meetings 1–6 days a week [13]. Jump outs are unofficial training activities where groups of horses ‘jump out’ from starting gates and gallop a set distance, used primarily to improve horses’ fitness through gallop training, or to educate young horses, and occur regionally once or twice a month. Trials are official mock races but without the pressure of result-driven outcomes, and are also hosted regionally once or twice a month. The aim of both is to gallop a horse under similar conditions to a race, and jockeys compete to obtain rides in both events, although they are not as financially important as race rides and have no weight restrictions for the jockey (catchweight). Distances are shorter, field sizes are smaller, and results are less important in jump outs and trials than races, with the emphasis on giving the horse the best ride possible, though jockeys are still expected to ride to their potential. In contrast, races are strictly controlled to ensure fair and competitive racing conditions where horses are allocated set weights to carry during a race, depending on the type of race and the experience and quality of the horse. Apprentices may claim a 1–4 kg reduction (allowance) on the assigned weight of the horse, to compensate for their inexperience in race riding. The amount of the allowance is reduced according to the number of winners the apprentice has ridden (thus can be used as a measure of experience). This allowance enables apprentices to obtain rides that otherwise may be preferentially offered to more experienced jockeys.

Understanding the sport’s competitive demands is the first step in building a specific training schedule to enhance performance in race riding. Training impulse (TRIMP) has been used as an integrative marker of exercise load undertaken by the athlete during training or competition and can be used as a comparative measure of exercise load [14]. For track work riding, TRIMP scores of 122 ± 67 for the entire session and 12 ± 6 per 5-min ride have been reported [12]. Energy expenditure for a jockey riding at canter has been reported as 7.1 ± 1.8 kcal∙min^−1^ corresponding to 7.7 metabolic equivalents (METs), lower than for jockeys in simulated races, at 17.5 ± 2.3 kcal∙min^−1^, and 9.4 METs [2,15]. The physical exertion experienced by jockeys in their daily riding efforts are lower and considered to be insufficient to prepare a jockey for the maximal physiological demands of riding in a race [15]. Therefore, a regular training schedule is important to meet the highly specific physical demands of race riding.

Whilst there is a plethora of information about horse performance, less is known about the physiological demands of jockeys, or how the jockeys’ body displacement and muscle activity can influence horse performance or jockey fatigue, and, ultimately, race performance, injury risk, and career longevity for jockeys during racing [2]. Most jockeys do not follow a structured fitness programme but instead rely on the regular horses’ training schedules (including riding trials) as preparation for racing [13,16]. Although the physiological demands and workload of jockeys riding in single races have been quantified, the demands on a jockey riding in multiple races and trials over the course of a single race day have not been examined.

Much of the study of jockey physiological parameters has been conducted during simulated races; however, it has been shown that jockeys work at a lower intensity and experience lower accelerations in simulated vs. competitive races [2,3,17]. However, inevitably, what few guidelines exist for jockey training rely mainly on data from simulated races. Additionally, to the authors’ knowledge, the muscle activity of jockeys has never been investigated in either simulated or competitive races, and may be a valuable tool in determining postural differences between training and racing, providing information for the formulation of future jockey training programmes. Jockey posture does vary between track work and races [12]; therefore, quantification and comparison of the relative exercise intensity demands of a jockey’s normal working schedule (racing preparation) are important to understand where and how a jockey-specific physical training programme could be implemented. Therefore, the aims of this study were to quantify the physiological demands of jockeys over the course of an entire trial or race day, describe jockey body displacements, and profile the muscular activity of jockeys riding in trials and races.

## 2. Materials and Methods

### 2.1. Participants

Eight apprentice (5 male and 3 female) and four (2 male and 2 female) senior jockeys holding a current and valid licence with the New Zealand Thoroughbred Racing (NZTR); the governing body for Thoroughbred racing in New Zealand, were recruited through NZTR. The apprentice jockeys held a current apprentice licence for 0.5–4 years and all had ridden in at least one race-day ride. The senior jockeys had ridden professionally for 14–38 years. It was not possible to collect race data from apprentice jockeys due to their extra weight constraints (riding 1–4 kg below the allocated weight for the horse). Jockeys in New Zealand have a relatively homogenous riding style, with apprentice school tutors reinforcing a uniform technique. The majority of jockeys in New Zealand tend to ride with their toe in the stirrup iron, rather than the full foot. Written informed consent was obtained prior to the commencement of data collection, and only jockeys over the age of 16 years were considered for the study. Ethical approval for this study was provided by the Institutional Human and Animal Ethics Committees.

### 2.2. Data Collection

Anthropometric data and predicted maximal aerobic capacity of the jockeys were assessed prior to (within 1 month) the commencement of field data collection. Stature was assessed to the nearest cm using a portable stadiometer (Seca 213, Hamburg, Germany). Body mass was measured in minimal light clothing (shorts and shirt) using portable digital weighing scales (Tanita InnerScan, Body Composition Monitor, BC-532). Body Mass Index (BMI) was calculated as the ratio of the weight to the square of height in metres (kg m^−2^). Participants performed the multistage 20 m shuttle test (beep test) [18] to determine their predicted maximal oxygen uptake (pred $\overset{\cdot }{V}O_{2\max}$).

Field data were collected from jockeys instrumented with a number of physiological monitoring devices (described below) at jump outs, trials, and races. Jump outs were grouped with trials and hereafter are labelled as trials. All horses ridden were entered in the trial or race as part of their normal race training or competition schedule. All devices attached to the jockey were synchronised to universal time and recorded continuously from the jockeys first ride to the completion of the last ride. Data from each device were downloaded after the completion of the day’s competition.

Speed, distance travelled, and heart rate (HR) of horse and jockey were determined using Polar human and equine HR monitors via Bluetooth to their respective watches (Polar V800 sports watch, Kempele, Finland) each containing a global positioning system (GPS) unit at a sampling rate of 1 Hz. Linear accelerations and displacements of horse and jockey were determined via synchronised wireless, tri-axial accelerometers with a reported accuracy of 0.0012 m s^2^ √Hz^−1^ (Emerald, APDM, Portland, OR, USA) and a sampling rate of 128 Hz, one attached to the horses’ girth and one to the back of the jockey’s helmet. Two additional accelerometers were attached to the jockey, one at the front of the jockey’s pelvis, close to their centre of mass (COM) and one centrally between the shoulder blades (upper body). These had a sampling rate of 1000 Hz, rectified and averaged to 25 Hz (Mytontec Muscle Monitor Version 3.1.1.3, Myontec Ltd, Kuopio, Finland). Horse HR and accelerations were measured only for horses racing in trials due to strict NZTR racing regulations prohibiting any interference with regulated horse equipment.

Skin surface electromyography (EMG) was recorded via a specialised set of tightly fitting elasticised textile clothing (Myontec Ltd, Kuopio, Finland) worn under the clothes the jockey normally wore whilst riding in trials and races. The clothing contained embedded electromyographic (EMG) electrodes that afforded measurement of skin surface EMG (µV) of eight muscle groups (*quadriceps*, *hamstrings*, *gluteal*, *erector spinae*/*lower back*, *abdominal external obliques*, *abdominal*, *trapezial* and *pectoral*) at a sampling rate of 1000 Hz, filtered with 40–200 Hz (−3 dB) band-pass filters, and digitalized with a 24-bit A/D converter and a Gain of 0. The 1000 Hz raw EMG signal was then rectified and averaged within the software programme (Mytontec Muscle Monitor Version 3.1.1.3, Myontec Ltd, Kuopio, Finland) to 25 Hz. The EMG electrodes were moistened with water before the jockey donned the clothing, to ensure adequate signal conduction. There were 2 sizes of EMG clothing, with the appropriate size matched to each jockey prior to data collection. The elasticised clothing was designed for a snug fit to conform to different body shapes and types, and, visually, there appeared to be good skin contact, especially with the stretched electrodes over the lower back area, the *quadriceps* and *hamstrings*. The clothing was worn with braces to minimise textile movement and increase skin contact with the electrodes over the upper body.

The time at which the jockey mounted, raced, and dismounted for each ride was manually recorded and subsequently matched to GPS data to identify the time of each race within the recorded data. Ride intensity was scored by the jockey after each ride using Borg’s rating of perceived exertion (RPE) 10-point scale [19], and horse temperament was assessed by the jockey with a “Horse Contact” 10-point scale, where 1 represented a horse that requires strong urging forwards, 3 represented a horse that is ‘on the bridle’ (easiest to ride) and 10 a horse that they are unable to stop (Table S1). The Horse Contact scale provided a simple measure of how tractable the horse was to ride, as this has been previously shown to affect the physiological demands on their jockeys [15,20].

### 2.3. Statistical Analysis

Data were initially scanned for obvious errors and then summarised using descriptive statistics. Data were presented as means ± SD unless otherwise stated. The normality of data distribution was tested using the Shapiro–Wilk test. Differences between races and trials, beginning and end of rides, displacement axes and first and last rides, were determined using Kruskal–Wallis and Wilcoxon rank sum tests for non-parametric data. One-and two-way repeated measures ANOVA comparisons were used to determine differences between displacements measured at different points on the jockeys’ bodies both within and between rides. Cohen’s effect size (ES) [21] was calculated to determine differences between the appropriate groups by calculating the difference between the means divided by the pooled standard deviation. The scale suggested by Hopkins, et al. [22] classes an effect size of 0.2 as a small effect, 0.6 as a moderate effect, 1.2 as a large effect, and 2.0 as a very large effect. TRIMP scores were calculated by summing the accumulated time (min) spent in five different HR zones according to Edwards [23]. HR_max_ est was estimated as 220—jockey age.

GPS data in combination with manually recorded data were used to determine the time spent riding or not riding. Riding time began when the jockey mounted the horse and finished when the jockey dismounted at the conclusion of the race, and consisted of a canter to the starting gates, racing, pulling up after the race, and returning to the parade ring. The trial or race portion was identified when the jockey was galloping at a velocity of ≥13.9 m∙s^−1^ [8,9], occurring < 10 s after leaving the starting gates, and allowed calculation of jockey variables whilst travelling at steady state gallop. Three 200 m sectionals (“Start”, “Middle” and “End”) were extracted from the corresponding portion of each trial or race (gallop). These sectionals were used to summarise accelerometer and EMG data. Non-riding time was the time spent between dismounting after each ride and mounting the next horse, and varied according to the individual jockey’s racing schedule.

Accelerometer data were filtered with a Butterworth low pass filter between 0.1–1.1 Hz to remove acceleration peaks. The magnitude of the acceleration vector was calculated as the sum of squares of the three movement planes. The dominant frequency (the highest magnitude sinusoidal component of the acceleration signal, corresponding to the horse’s stride frequency) of the cyclical movement of the horse and jockey was determined by analysing the results of the Fast Fourier Transform of the acceleration signal. Acceleration data were doubly integrated to quantify displacement, following published methods [24]. The delay of the time of the jockey and horse’s point of vertical acceleration was measured for each stride, averaging the time differences between the acceleration minima over a 200 m sectional.

EMG signal power and dominant frequency were determined by Fourier Transform. Muscle activity was calculated by taking the mean signal recorded for each individual muscle as a percentage of the total signal recorded from all muscles investigated over the 200 m sectional. The left/right side balance was calculated as the percentage of left side muscle signal of the total signal for each muscle group (excluding *Abdominals* which was recorded across the centre of the muscle group). These methods were used in preference to the typical method of normalising EMG signal to a percentage of maximum voluntary contraction, due to previous research suggesting that the typical method is inappropriate to describe the quasi-isometric riding position [6]. Additionally, the generalised EMG signal enabled only gross comparison between muscle groups, allowing the riding position in training, trials, and races to be characterised, rather than specific analysis of each muscle group.

All statistical analyses were conducted using RStudio (version 3.5.1, 2018; R Foundation for Statistical Computing, Vienna, Austria) with the level of significance set at *p* < 0.05.

## 3. Results

Descriptive and mean anthropometric data for eight apprentice and four senior jockeys are presented in Table 1. Predicted $\overset{\cdot }{V}O_{2\max}$ was assessed for all apprentice jockeys and two senior jockeys.

Race data were collected for senior jockeys only and complied with all NZTR racing regulations. Trial data were collected for all apprentice and two senior jockeys. Ridden horses included both male and female Thoroughbred horses in active race training, ranging from 2–10 years old, and were entered in the trial or race on the day of data collection. Sixty-eight separate rides were recorded with 68 different horses. Of these, 52/68 rides were trials, and 16/68 rides were races. GPS and jockey HR data were recorded for *n* = 43/52 trials and *n* = 16/16 race rides. HR data for the horses were recorded for 7/52 trials. Accelerometer data for the horses were recorded for *n* = 32/52 trials. Accelerometer data for the jockey’s head were recorded for *n* = 48/52 trials and *n* = 10/16 race rides. EMG and accelerometer data for the jockey’s upper body and COM were recorded for 48/52 trial rides and 16/16 race rides.

The jockeys rode a mean (± SD) of 5 ± 4 horses in trials and 4 ± 2 horses in the races. The jockeys’ time allocation for trials and races and their corresponding HRs are shown in Table 2. Jockeys had less non-riding time between successive trial rides than in the races, with an average of 18.1 ± 22.6 min between rides in the trials (ranging from 0.5–94.1 min) and 38 ± 17 min (ranging from 24–66 min) between rides in the races (*p* < 0.001). The average HR of jockeys riding in a trial was ~81% HR_max_ and was ~60% HR_max_ for the entire time spent at the trials. The average HR of jockeys riding in a race was ~93% HR_max_ and was ~64% HR_max_ for the entire time spent in the races. The average (± SD) TRIMP score for the entire time spent by a jockey riding in trials was 200 ± 79 and 292 ± 106 in the races (*p* = 0.1). Mean horse HR_peak_ during a trial (*n* = 7) was 203 ± 22 bpm.

The characteristics of work done by the horse and jockey (physiological response) per trial or race ride are shown in Table 3. Horses galloped faster in trials (*p* < 0.05) over shorter distances (ranging from 800–2200 m) than in races (ranging from 1200–2200 m, *p* < 0.01). Jockey HRs as percentages of estimated HR_max_ and TRIMP scores in trials were lower than for jockeys riding in a race (*p* < 0.001). The average RPE reported for a trial (3.6 ± 1.9, corresponding to moderate/somewhat hard intensity) was less than for a race (7.9 ± 2.4, corresponding to very hard intensity, *p* < 0.001), with no difference in RPE reported between the first and last ride of the day (*p* = 0.4 trials, *p* = 0.2 races). Horse Contact was 3.8 ± 2.2 for trial rides and 3.6 ± 2.0 for race rides (*p* = 0.4), both corresponding to marginal contact.

### Accelerations and Muscle Activity

The dominant frequency (stride frequency) of the horse movement in trials was consistent in all planes at 2.3 ± 0.4 Hz. Jockey dominant frequency matched the horse in both races and trials at 2.3 ± 0.1 Hz (*p* = 1) and was consistent in all planes. Jockey movement preceded horse movement by 0.027 ± 0.051 s in trials. Jockeys experienced lower mean accelerations (16.1 ± 5.6 m∙s^−2^ in trials and 15.8 ± 5.2 m∙s^−2^ in races) and peak accelerations (25.3 ± 8.4 m∙s^−2^ in trials and 28.1 ± 12.6 m∙s^−2^ in races) than the horses (mean 37.5 ± 8.4 m∙s^−2^, peak 68.9 ± 11.3 m∙s^−2^ in trials, *p* < 0.001). Jockey acceleration in the vertical plane was approximately twice that of the medial/lateral and fore/aft planes in both trials and races (Table S2).

**Figure 1:**
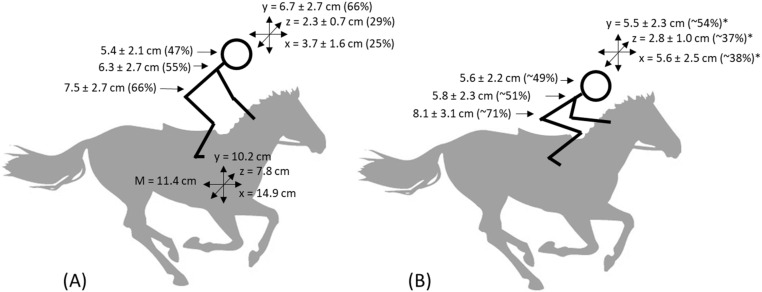
Mean magnitude (M) of displacements (cm) and relative displacement (%) of jockey to a horse galloping in (**A**) Trials and (**B**) Races, and in the vertical (y), fore/aft (x), and medio/lateral (z) planes. Horse displacements were measured only for horses racing in trials. All differences between jockey and horse (at trials) displacement are significant (*p* < 0.05). * Denotes differences between jockey displacement in trials and races (*p* < 0.05).

The mean displacements and approximate riding position of the jockey at trials and races are shown in Figure 1. Displacements of jockeys riding in both trials and races were smaller than the displacement of a horse galloping at trials in all areas and planes (*p* < 0.001). Jockey head displacement was smaller in the vertical plane and larger in the medio/lateral and fore/aft planes for jockeys riding in races than in trials. The magnitude of jockey displacement in trials decreased from COM to neck to head (*p* < 0.001) with Cohen’s effect size decreasing from very large 2.0 (horse—jockey COM) to moderate 0.6 (jockey COM—upper body) to small/moderate 0.5 (jockey upper body—head). In races, jockey COM displacement was greater than displacement at the upper body and head (*p* < 0.001). Cohen’s effect size was large, at 1.2, between the jockeys’ COM and neck, with no difference (ES = 0.1, *p* = 0.7) between jockey upper body and head displacement in races.

Jockey COM displacement decreased within a trial ride. Jockey head displacement increased within both trial and race rides (Table 4). The difference in movement between jockey COM and head decreased within both a trial and race ride. Jockey COM and head displacement at the end of a race ride was greater than at the end of a trial ride.

In both trials and races, the jockeys’ quadriceps muscle group was responsible for one-quarter of the total muscle activity (Table 5). In trials, muscle activity was distributed evenly between the jockeys’ legs (*quadriceps*, *hamstrings* and *gluteal*, 36%) and ‘core’ musculature (*lower back*, *obliques* and *abdominals*, 39%), with upper arm muscles (*trapezius* and *pectorals*, 26%) contributing one-quarter of the total muscle activity. This distribution differed for jockeys riding in races, with half (51%) of total muscle activity in the jockeys’ legs, with ‘core’ muscles contributing 35%, and upper arm muscles only 14% of total muscle activity. The balance of muscle activity between the left and right sides of muscle groups was relatively even for jockeys at trials, with *obliques* and *trapezius* having a slight left bias. In trials, the lower body muscles (*quadriceps*, *hamstrings*, *gluteal*) were activated at a similar dominant frequency as the horse movement, whereas the lower back and upper body muscles had more variable dominant frequencies. In races, the *hamstrings* had a left side bias, and all jockey muscles had variable dominant frequencies.

The percentage of muscle activity of the jockey *trapezius* muscle group increased between the first and last trial ride, and *abdominal* activity decreased between the first and last race ride (Table 6). Both within and between rides for each race type, there was little change in the distribution of jockey muscle activity. Upper arm muscles (*trapezius* and *pectorals*) contributed less and *hamstrings* more to overall muscle activity in all sectionals of race rides than in trial rides.

## 4. Discussion

To the authors’ knowledge, this is the first study to quantify the physiological demands, describe jockey body displacements, and profile the muscle activity of jockeys riding in both trials and races. In trials, which lasted 2 h on average and involved multiple rides, jockeys had high HRs and exercised at a moderate to high aerobic intensity. In contrast, during a race day, which lasted 2.5 h on average and also involved multiple rides, jockeys had near maximal HRs and near maximal aerobic demand during a race. In trial riding, jockeys adopted a crouched posture, using their legs to dampen horse oscillation using core and upper body muscles for postural control, with increased reliance on the arms for postural support after multiple rides. Jockey displacement was damped throughout the body, with medio/lateral and fore/aft movement of the head minimised. In contrast, during races, jockeys adopted a lower crouched posture, requiring increased *hamstring* activity to support their COM positioned anteriorly, damping horse movement mainly through both knee and hip, flexion and extension. Vertical displacement of the jockeys’ heads was lower and fore/aft displacement higher than for trials. In essence, they used their entire body for concurrent damping, postural control, and to urge the horse to greater speeds.

Since the mean age of the senior (race riding) jockeys was twice that of the apprentice jockeys, relative measures of HR were used and are considered to provide a valid comparison method between trial and race rides. In races, the jockeys exercised at a higher intensity than in trials. Their relative mean and peak HRs were similar to those previously reported in jockeys riding in races in Ireland, Hong Kong, and the United States [2,3,17,25]. This finding was reflected in the TRIMP scores, which were twice as high during races than for trials. For the duration of the event (all the comparable durations), the TRIMP of a jockey riding in the races (292 ± 106) was higher than in the trials and over twice that of track work riding (122 TRIMP) [12]. Comparatively, the exercise load of a professional soccer player in a game lasting 1.5 h is ~190 TRIMP and for an athlete in a world-class marathon race (~2 h) is ~275 TRIMP [14], though differences in sport-specific variables such as eccentric muscular contractions are not taken into account in this measure. Between successive races, there was some opportunity for the jockeys to rest and recover (25–30 min between races). This time was spent speaking to trainers and preparing equipment to ride the next horse, requiring a low cardiovascular challenge, and may be a potential area to investigate rest and recovery strategies of experienced and novice jockeys. Thus, during a race meeting, jockeys experienced intermittent periods of intense cardiovascular load with sustained periods of elevated heart rate, similar to that observed in harness racing drivers [26]. Between trials, the changeover between successive rides could be less than 1 min, allowing little rest and recovery time between rides.

A jockey is required to balance and control the horse during the race, and horse temperament has been suggested as a contributor to the jockey’s workload [1,12,27]. However, in the present study, jockeys reported similar variations in horse contact during trials and races, indicating that when operating at maximal or near maximal capacity (both jockey and horse), horse temperament was not a significant factor in jockey workload. Horse contact is only one indicator of the temperament of the horse, so other behavioural indicators (such as a measure of ‘fractious’ behaviour’) may be worth considering in future studies.

Jockey HR may have a psychological component, particularly in their non-riding time. Pressure from owners and trainers to perform well in a race, as well as personal pressure to obtain financial rewards and career opportunities, may all contribute to the elevated HR observed between races. Inspection of the jockey HR curve during the race showed that HR increased throughout the race, and peaked after race completion, implying that during a trial or race, jockey racing HRs were physiologically driven, rather than by an adrenal or stress response to the pressure of competition. Despite the high cardiovascular loads during trials and especially during races, most jockeys do not follow a structured fitness programme but rely on regular track work and riding horses in trials as preparation for racing [13,16].

Synchronous muscle activity throughout the jockey’s body damped external oscillation (horse movement), minimising movement of the jockey’s head. An analogous pattern of muscle activity has previously been observed in track-work riders [12] and cross-country mountain bikers [28,29]. In trials, head stability in the frontal plane was maintained, with 3–4 times less movement of the head than the horse in the medio/lateral and fore/aft planes. The magnitude of displacement of the jockey was greatest at their COM, decreasing towards their upper body and head, similar to jockeys riding track work [12]. This was achieved by activating leg muscles (*quadriceps*, *hamstrings* and *gluteal*) in synchrony with the horse’s movements. The lower dominant frequency of the ‘core’ muscles (*lower back*, *obliques* and *abdominals*) indicated their use in maintaining the postural stability of the jockey, with the upper arm muscles (*trapezius* and *pectorals*) in contact with the horse’s neck aiding in the stabilisation of the jockey on the horse. Although there was little change in muscle group distribution of the jockey in a trial ride, their head movement increased, indicating that they may have had less control over the synchronous activity of their muscles towards the end of the ride, resulting in less damping of horse movement—a possible indication of neuromuscular fatigue. The increase in *trapezius* activity between the first and last trial ride could indicate that jockeys became more reliant on using their hands on the horses’ necks after riding multiple trial rides, another indication of the jockey feasibly mitigating the effects of fatigue. In contrast to track work riding [12], jockey movement in trials preceded that of the horse, indicating the jockey was moving more in synchrony with the horse and anticipating horse movement, as has been observed in more skilled dressage riders compared to novices [30,31,32].

The position of the jockey was subtly different between trials and races. The differences were pictorially exaggerated for clarity in Figure 1. The displacement of the jockey’s head in the vertical plane in race riding jockeys was lower than observed for jockeys riding in trials, indicating that the crouch posture of jockeys in a race was lower than for trials. This finding is consistent with the observation that, in vibration studies, smaller knee angles have been associated with reduced head accelerations [33]. The muscular activation profile of jockeys in races had a higher proportion of *hamstring* activity than that of jockeys riding in trials. The higher *hamstring* activation in race riding may be a result of the lower ‘crouched’ position resulting in longer muscle length causing greater activation of the *hamstring* muscle group to concurrently support jockey COM and damp horse motion. This indicates that jockeys in races balanced on their toes, closing their hip and knee angles, with their COM lowered and shifted anteriorly. The larger ES (1.2) observed between the race riding jockey’s COM and upper body displacement support the idea that horse movement was damped mainly by knee and hip—flexion and extension, controlled by the lower legs of the jockey in contact with the sides of the horse. This contrasts with a trial rider, having a wider knee and hip angle, maintaining their COM and upper body more centrally over their feet, with a smaller difference between COM and upper body movement (ES = 0.6), similar to that observed in track-work jockeys [12] and equestrian riding in the two-point position [34]. This position would require less *hamstring* activity and less physiological (aerobic) work to maintain [35]. Additionally, trial-riding jockeys may use their arms on the horses’ neck to stabilise themselves, like the position adopted during track work [12], reducing the reliance on the *hamstrings*.

The magnitude of displacement of the race jockeys was greatest at their COM. This was similar for jockeys riding in track work and trials, with additional damping occurring between the COM and upper body, but for race jockeys, there was no damping between the upper body and head. The posture of jockeys during races allowed greater damping of vertical horse oscillation but resulted in greater head movement in the medio/lateral and fore/aft planes than observed in trial riding jockeys. Thus, less work is required by the horse to move the mass of the jockey vertically through each stride cycle, potentially allowing the horse to achieve greater speeds [36]. The weight distribution of a rider is an important aspect in the ability of a horse to move freely and easily [37] and race jockeys appeared to have their weight positioned more anteriorly than trial and track-work jockeys, which perhaps allowed the horse to move more freely. Jockey head displacement in the fore/aft plane during races was 50% more than that of a jockey riding in trials, and was likely due to the jockey enhancing momentum gains in optimised oscillations associated with their weight displacement, to positively encourage the horse to increase speed in the ‘push’ to the finish line.

Race riding jockeys used their arms and hands solely for directional control and urging the horse forward, resulting in a smaller proportion of upper arm muscle activity compared to trial riding jockeys. The dominant frequencies of oscillation for all muscle groups of the jockeys were lower than those estimated for the horses. This indicated that muscles throughout a jockey’s whole body were not only responsible for postural damping of movement, but also functioned independently of the horse’s movement, as the jockey both anticipated and influenced horse movement. This may have been a result of the jockeys racing strategy, whereby they were constantly adjusting the horse’s pace and position in the race to optimise their opportunity to race to their maximum potential. Jockey head movement increased within a race. This may have been an indication of fatigue, resulting in less effective synchronous muscle activation, but was likely confounded by the additional activity of the jockey checking their position and urging the horse forwards in the final stages of the race. *Abdominal* activity was halved between the first and last race ride, indicating that this muscle group may have become fatigued in race riding jockeys, resulting in postural changes possibly reducing their riding effectiveness.

Track work riding has been previously shown to demand a low to moderate physiological response in jockeys [12,15]. The present study has shown that trial riding demands a higher cardiovascular and muscular response, with race riding requiring maximal exertion. Thus, trial riding appears to act as a segue between track riding and race riding, even though the jockey’s posture is similar for both trial and track work riding. Since trial riding requires less of the same physical exertion and a different posture than that adopted by jockeys during a race, trial riding is likely to be insufficient preparation for race riding. Acquisition of both trial and race rides is largely dependent on a jockey’s skill and performance record, suggesting that apprentice jockeys may not be provided with ample opportunities to ‘practice’ and achieve race fitness. Indeed, using races themselves to acquire fitness implies that those jockeys are inadequately fit for racing, and the jockey would thus not be performing to their best potential at this highest level of competition. Jockeys who ride in races regularly and are assumed to be ‘race fit’ have greater success and lower injury risk (from falling) than the majority of jockeys who struggle to obtain multiple race rides [38]. Lower fitness has been associated with a higher risk of fall and injury in jockeys [39], and the suggestion of fatigue in race jockeys could contribute to the higher fall risk seen in jockeys with few race rides.

Advanced equestrian riders have a higher ability to anticipate horse movement at a neuromuscular level, with more defined and coordinated muscular activation patterns than novice riders, resulting in greater rider stability and synchronisation with the horse, which, in turn is associated with a lower injury risk to both horse and rider [5,30,40,41]. A lack of synchronicity additionally results in greater energy expenditure in less experienced riders [42]. Jockeys require a high level of baseline aerobic and anaerobic fitness, in addition to targeted exercises to improve neuromuscular control and coordination, to improve and maintain on-horse stability and control. By improving the conditioning of jockeys, it is hypothesised that riding performance can be enhanced, and the opportunity for jockeys to positively influence horses in race situations will increase. Therefore, the importance of sufficient race fitness for jockeys is not only to improve horse performance and welfare, but also jockey safety and the longevity of their career. The difference in posture and the maximal physiological demands required by jockeys to perform in a race indicate that specific off-horse physical preparation to mimic the repeated high-intensity demands of race riding would be beneficial, especially to jockeys who are beginning their career, or who are rehabilitating or unable to acquire sufficient rides to achieve and maintain a racing level fitness.

### Limitations

Attempting to collect data in the real world is fraught with difficulty, which resulted in an imbalanced study design, with apprentice jockeys clustered in the trial dataset and only senior jockeys in the race dataset. Due to the weight allowances of apprentice jockeys, these jockeys are under considerably more pressure to meet lower weight limits assigned to horses on race day. Participation in this study was voluntary and relied on good will, as jockeys were not contracted to participate in this trial. Therefore, data acquisition was dependant on the commercial reality of a jockeys’ livelihood which was their primary consideration, rather than risking exceeding a weight limit and losing a ride. The difference in jockey experience between the two datasets was a confounder; however, there were no obvious differences in the magnitude or direction of displacements and EMG data between jockeys and apprentices within the trial data. The small number of jockeys within the trial dataset precluded logical statistical analysis within that dataset. However, based on the lack of differentiation between jockeys and apprentices during trial riding, it would be unlikely that the differences between race and trial riding are solely due to jockey age or experience, given the obvious postural differences between trial and race riding.

The regulations of racing prohibit any intervention which may alter the outcome of the race. As such, monitoring of the participants was restricted to non-invasive or indirect measures. In flat racing, jockey weight is a method of handicapping horses, thus all equipment was required to be small, lightweight, and unobtrusive to the jockey. This allowed the jockey to make the correct weight assigned to the horse as well as ensuring unrestricted movement, guaranteeing that there was no interference with the jockey’s capability to control the horse or limit final race placing. Additionally, jockey whip use, particularly in the final stages of the race, may confound results, perhaps resulting in greater jockey postural displacement or COM deviations. In New Zealand, the use of the whip is restricted to no more than five times prior to the final 100 m, after which it can be used at the jockey’s discretion. However, there is tight judicial control over excessive use of the whip, which effectively limits jockey medio/lateral displacements due to whip use in the final straight. In New Zealand, it is common practice for jockeys to ride with only their toes in the stirrup iron, with the irons almost parallel to the side of the horse. Differences in foot placement in the stirrup may provide limited variation in the ability to attenuate displacement. However, the primary mechanism for damping horse movement is due to muscular activation of the jockey’s proximal limb.

Whilst HR is a common means of monitoring physical activity intensity, it cannot measure metabolic rate, and thus the physical work requirement. Nevertheless, it does provide a good indicator of cardio-respiratory and vascular stress and, in the absence of any other measures, provides some insight into the physiological requirements of the sport and demands on the jockey. Measuring the energy expenditure (as an indication of aerobic demands) and power output (as a measure of anaerobic demands) of jockeys and horses, and how it could be used to determine optimal training as in other sports (cycling, rowing, running, kayaking) could be an interesting future avenue for study.

Both trials and races varied in distance, but on average races were longer than trials, which likely accounted for the higher speeds observed in trials than races. Thus, a comparison of middle and end sectionals of an 850 m trial with a 2200 m race may not be valid. It has been reported that the mean HR of a jockey in a long flat race (2313 ± 142 m) was lower than in a shorter flat race (1247 ± 185 m), but that there were no differences in peak HR, RPE, or blood lactate concentrations [3]. Considering the difficulty in capturing data from multiple competitive rides, separating the results to compare races and trials of similar distances was not feasible in the present study.

## 5. Conclusions

Jockeys riding in trials and races experienced long periods (2–3 h) of low–moderate intensity cardiovascular demand interspersed with periods of high or maximal physiological effort. Trial riding jockeys exercised at a lower intensity than race riding jockeys and activated their legs and arms to support and dampen horse oscillation, using ‘core’ musculature to maintain postural stability and minimise head displacement in the medio/lateral and fore/aft planes. Jockeys riding in races exercised near maximally in a lower crouched position, with a higher proportion of *hamstring* activation with their COM positioned anteriorly, using their legs, ‘core’, and upper body to both damp the oscillations and control the horse. This position resulted in lower vertical displacement of the head, but greater displacement in the medio/lateral and fore/aft planes than a trial riding jockey. The differences in cardiovascular demand and riding posture in jockeys riding in trials and competitive races indicate that there is a need for an off-horse jockey-specific physical training programme to improve jockeys’ race performance, which may be beneficial to jockeys’ overall riding safety and career prospects. Greater jockey stability and coordination will in turn have mutual benefits for the horses’ welfare and performance, ensuring both athletes perform to their maximum potential, and enabling greater opportunity for jockeys to positively influence horses in race situations. Further research comparing jockey postural deviations and muscle activity with jockey experience, performance, and after specific exercises may inform future assessment criteria for the development of safe and successful jockey education programmes in the racing industry.

## Figures and Tables

**Table 1:** Descriptive data (mean ± SD) for apprentice (*n* = 8) and senior (*n* = 4) jockeys.

Variable	Apprentice	Senior	All
Age (yrs)	23 ± 3	41 ± 10	29 ± 10
Height (cm)	162.1 ± 5.4	159.9 ± 7.1	161.4 ± 5.4
Body Mass (kg)	51.5 ± 2.0	52.6 ± 1.9	51.9 ± 2.1
BMI (kg∙m^−2^)	20.1 ± 0.9	20.7 ± 1.2	20.1 ± 0.9
Predicted $\overset{\cdot }{V}O_{2\max}$ (mL∙kg^−1^∙min^−1^)	45.1 ± 6.6	42.8 ± 6.4	44.6 ± 6.7

**Table 2:** Time allocation and HR (mean ± SD) during a day at the trials (*n* = 10) or races (*n* = 4) for 12 jockeys.

Activity	Trials		Races	
	Time (min)	HR Average (bpm)	Time (min)	HR Average (bpm)
Entire Trials/Races	125 ± 58	119 ± 17	153 ± 94	114 ± 17
Non-riding	71 ± 43	115 ± 23	102 ± 73	98 ± 11
Riding	54 ± 20	140 ± 15	52 ± 23	130 ± 10
Trial/Race	5.5 ± 2.8	160 ± 17	6.1 ± 2.3	166 ± 10

**Table 3:** Descriptive data (mean ± SD) of the characteristics of the trial (*n* = 52) and race (*n* = 16) rides and corresponding jockey physiological response per ride.

Descriptive (Horse) Variables	Trials				Races				
	Start	Middle	End	Entire Ride	Start	Middle	End	Entire Ride	ES ^+^
Time (s)	11.6 ± 0.8 *^,a^	12.0 ± 1.0 *^,b^	12.4 ± 0.7 *^,c^	69.2 ± 19.8 *	12.1 ± 0.3 ^a^	12.7± 0.8 ^b^	13.0 ± 0.5 ^b^	91.4 ± 22.6	1.5
Distance (m)	200 ± 5 *	200 ± 5	200 ± 4	1135 ± 276 *	206 ± 3 ^a^	201 ± 4 ^b^	200 ± 5 ^b^	1449 ± 303	1.5
Velocity (m∙s^−1^)	17.4 ± 1.1 *^,a^	16.8 ± 1.2 *^,b^	16.2 ± 0.8 *^,c^	16.5 ± 0.8 *	17.1 ± 0.4 ^a^	15.9 ± 0.9 ^b^	15.4 ± 0.5 ^b^	16.0 ± 0.6	1.0
Stride count	27 ± 1 ^a^	28 ± 2 ^b^	29 ± 2 ^b^	-	28 ± 2 ^a^	29 ± 2 ^b^	29 ± 2 ^b^	-	
Stride length (m)	7.4 ± 0.4 ^a^	7.1 ± 0.4 ^b^	7.0 ± 0.4 ^b^	-	7.5 ± 0.6 ^a^	6.9 ± 0.4 ^b^	6.8 ± 0.3 ^b^	-	
Stride duration (s)	0.43 ± 0.02 ^ab^	0.42 ± 0.02 *^,a^	0.43 ± 0.02 ^b^	-	0.44 ± 0.03	0.43 ± 0.02	0.44± 0.02	-	
**HR variables**									
Mean HR (bpm)	149 ± 19 ^a^	162 ± 18 ^b^	172 ± 19 ^c^	160 ± 17	151 ± 11 ^a^	167 ± 11 ^b^	178 ± 12 ^c^	166 ± 10	0.6
Relative mean HR (%HR_max_ est)	76 ± 10% *^,a^	83 ± 10% *^,b^	87 ± 11% *^,c^	81 ± 9% *	85 ± 3% ^a^	94 ± 3% ^b^	101 ± 3% ^c^	94 ± 2%	2.5
Peak HR (bpm)	153 ± 20 ^a^	167 ± 18 ^b^	175 ± 18 ^c^	177 ± 16	156 ± 12 ^a^	170 ± 11 ^b^	180 ± 11 ^c^	182 ± 10	0.5
Relative peak HR (% HR_max_ est)	78 ± 11% *^,a^	85 ± 10% *^,b^	89 ± 9% *^,c^	90 ± 9% *	88 ± 3% ^a^	96 ± 2% ^b^	102 ± 3% ^c^	102 ± 2%	2.6
TRIMP	0.65 ± 0.21 *^,a^	0.83 ± 0.22 *^,b^	0.96 ± 0.21 *^,c^	4.42 ± 1.80 *	0.89 ± 0.07 ^a^	1.12 ± 0.10 ^b^	1.16 ± 0.05 ^b^	7.21 ± 1.83	2.2

^+^ Effect Size (ES) between race and trial entire ride. Means differ (*p* < 0.05) between trial and race sectionals (*) and across rows (^abc^) within each race type.

**Table 4:** Mean (± SD) magnitude of displacements (m) of horse and jockey in a trial (*n* = 48) or race (*n* = 16) and between the first and last ride (*n* = 10, trials, *n* = 4 races). Effect sizes (ES) are between horse displacement at trials and jockeys’ head, or start and end sectionals.

Riding Type	Position	Within Ride				Between Rides		
		Start	Middle	End	ES	First Ride	Last Ride	ES
Trials	Horse	0.110 ± 0.028 ^1^	0.112 ± 0.029 ^1^	0.121 ± 0.025 ^1^	0.6	0.104 ± 0.025 ^1,a^	0.124 ± 0.024 ^1,b^	1.2
	Jockey COM	0.085 ± 0.039 ^2,a^	0.072 ± 0.017 ^2,ab^	0.068 ± 0.016 ^23,b^	0.8	0.075 ± 0.048 ^2^	0.079 ± 0.025 ^2^	0.1
	Jockey upper body	0.061 ± 0.035 ^3,ab^	0.061 ± 0.020 ^3,a^	0.068 ± 0.024 ^2,b^	0.3	0.066 ± 0.041 ^2^	0.067 ± 0.024 ^2^	0.0
	Jockey head	0.043 ± 0.015 ^4,a^	0.058 ± 0.021 ^3,b^	0.061 ± 0.023 ^3,b^	1.3	0.058 ± 0.029 ^2^	0.058 ± 0.022 ^3^	0.0
	Δ (horse—head)	0.067 ± 0.025	0.057 ± 0.031	0.063 ± 0.028	0.2	0.054 ± 0.031	0.065 ± 0.024	0.6
	Δ (COM—head)	0.043 ± 0.041 ^a^	0.015 ± 0.027 ^b^	0.007 ± 0.021 ^b^	1.6	0.017 ± 0.056	0.024 ± 0.029	0.2
	ES (horse to head)	4.2	3.0	3.5		2.4	4.1	
Races	Jockey COM	0.082 ± 0.048 ^1^	0.076 ± 0.012 ^1^	0.085 ± 0.021 *	0.1	0.095 ± 0.054 ^1^	0.080 ± 0.020	0.5
	Jockey upper body	0.047 ± 0.024 ^2,a^	0.048 ± 0.012 ^2,a^	0.078 ± 0.018 ^b^	2.1	0.064 ± 0.030 ^2^	0.058 ± 0.023	0.3
	Jockey head	0.036 ± 0.004 ^12,a^	0.052 ± 0.013 ^2,b^	0.080 ± 0.017 ^c,^*	5.0	0.056 ± 0.021 ^12^	0.067 ± 0.029	0.6
	Δ (COM—head)	0.062 ± 0.019 ^a^	0.021 ± 0.007 ^ab^	0.016 ± 0.005 ^b^	4.7	0.047 ± 0.085	0.019 ± 0.026	0.6
	ES (horse to head)	~5.2	~3.8	~2.7		~2.9	~3.0	

Means with differing superscripts differ (*p* < 0.05) down columns within each riding type (^123^), across rows (^abc^) and between jockey riding at trials and races (*).

**Table 5:** Mean (± SD) surface EMG parameters of eight muscle groups for jockeys (*n* = 12) riding in trials (*n* = 48) and races (*n* = 16).

Muscle Group	Trials			Races		
	Muscle Activity (%)	Left/Right Side Balance (% Left Side)	Dominant Frequency (Hz)	Muscle Activity (%)	Left/Right Side Balance (% Left Side)	Dominant Frequency (Hz)
*Quadriceps*	23 ± 10 ^a^	49 ± 9 ^a^	2.0 ± 0.6 ^a^	26 ± 13 ^a^	51 ± 8 ^a^	1.8 ± 0.7 ^a,^*
*Hamstrings*	7 ± 4 ^b^	49 ± 9 ^ab^	2.0 ± 0.6 ^a^	21 ± 15 ^ac,^*	64 ± 15 ^b,^*	1.3 ± 0.8 ^ab,^*
*Gluteal*	6 ± 4 ^c^	46 ± 9 ^bd^	2.0 ± 0.7 ^a^	4 ± 3 ^b,^*	49 ± 13 ^ac^	1.5 ± 0.8 ^ab,^*
*Lower back*	15 ± 9 ^d^	51 ± 10 ^ac^	1.6 ± 0.8 ^bc^	19 ± 11 ^c,^*	51 ± 9 ^a^	1.6 ± 0.8 ^ab^
*Obliques*	16 ± 9 ^d^	53 ± 13 ^ce^	1.5 ± 0.7 ^b^	11 ± 6 ^d,^*	49 ± 8 ^ac,^*	1.3 ± 0.8 ^ab^
*Abdominals*	8 ± 6 ^b^	-	1.5 ± 1.0 ^bc^	5 ± 3 ^b,^*	-	1.4 ± 0.9 ^ab^
*Trapezius*	12 ± 9 ^e^	54 ± 13 ^e^	1.7 ± 0.8 ^bc^	5 ± 5 ^b,^*	45 ± 9 ^c,^*	1.3 ± 0.9 ^ab,^*
*Pectoralis*	14 ± 7 ^d^	49 ± 9 ^ad^	1.8 ± 0.7 ^ac^	9 ± 5 ^d,^*	53 ± 10 ^a^	1.2 ± 0.8 ^b,^*

^abcde^ Values with different superscripts differ significantly within columns (*p* < 0.05) and between jockeys riding in trials and races (*).

**Table 6:** Mean (± SD) muscle activity (%) of jockeys within a ride (*n* = 48 trials, *n* = 16 races) and between the first and last ride during trials (*n* = 48) and races (*n* = 16). Effect sizes are between the first and last sectional of the ride.

Riding Type	Muscle Group	Start	Middle	End	ES	First Ride	Last Ride	ES
Trials	*Quadriceps*	21 ± 8	23 ± 10	25 ± 11	0.7	25 ± 10	22 ± 10	0.4
	*Hamstrings*	7 ± 4	7 ± 4	7 ± 3	0.1	8 ± 5	7 ± 3	0.4
	*Gluteal*	6 ± 3	6 ± 3	6 ± 6	0.0	5 ± 2	5 ± 3	0.3
	*Lower back*	17 ± 10	15 ± 9	13 ± 7	0.6	15 ± 8	17 ± 12	0.3
	*Obliques*	16 ± 9	16 ± 9	15 ± 8	0.1	17 ± 11	15 ± 6	0.3
	*Abdominals*	8 ± 6	8 ± 5	7 ± 6	0.2	8 ± 8	10 ± 7	0.3
	*Trapezius*	10 ± 8	11 ± 9	13 ± 10	0.4	9 ± 3 ^a^	12 ± 6 ^b^	1.1
	*Pectoralis*	15 ± 7	14 ± 7	13 ± 6	0.5	14 ± 7	12 ± 4	0.5
Races	*Quadriceps*	27 ± 14	27 ± 14	24 ± 12	0.3	25 ± 13	24 ± 7	0.1
	*Hamstrings*	25 ± 17 *	20 ± 15 *	18 ± 13 *	0.6	15 ± 11 *	31 ± 18 *	1.5
	*Gluteal*	4 ± 3	5 ± 3	4 ± 3	0.2	4 ± 2	3 ± 3 *	0.2
	*Lower back*	19 ± 12	19 ± 13	20 ± 10 *	0.2	21 ± 11 *	21 ± 17	0.0
	*Obliques*	9 ± 6 *	11 ± 5 *	13 ± 6	0.8	11 ± 6	8 ± 3 *	0.9
	*Abdominals*	4 ± 3 *	6 ± 3	6 ± 4	0.8	8 ± 4 ^a^	3 ± 2 ^b,^*	2.0
	*Trapezius*	4 ± 5 *	5 ± 4 *	7 ± 5 *	0.7	8 ± 7	3 ± 3 *	1.2
	*Pectoralis*	8 ± 5 *	9 ± 5 *	9 ± 5 *	0.3	9 ± 5 *	6 ± 2 *	0.9

^ab^ Values with different superscripts differ significantly across rows (*p* < 0.05) and between jockeys riding at trials and races (*).

## Data Availability

Data is available on request from the authors.

## Supplementary Materials

The following supporting information can be downloaded at: https://www.mdpi.com/article/10.3390/ani12182351/s1, Table S1: Horse contact rating scale and Borg’s rating of perceived exertion 10-point scale used with jockeys (n = 10) to score each ride of a morning’s track-work.; Table S2: Mean (± SD) linear accelerations and displacements of horse (n = 32) and jockey’s head during trials (n = 48) and races (n = 10).
