# Supplementary material for: Physiological Demands and Muscle Activity of Jockeys in Trial and Race Riding
Source: Animals (Basel). 2022 Sep 8;12(18):2351. doi: 10.3390/ani12182351 (PMC9495223; doi:10.3390/ani12182351)
Supplement: Supplementary file 1 [file animals-12-02351-s001.zip › Table S2.pdf]

Table S2. Mean (± SD) linear accelerations and displacements of horse (n = 32) and jockey's head during trials (n = 48) and races (n = 10).

Variable	Descriptor	Vertical	Medio/lateral	Fore/Aft	Magnitude
Dominant frequency (Hz)	Trials Horse	2.3 ± 0.2	2.4 ± 0.1	2.3 ± 0.2	2.3 ± 0.4
	Trials Jockey	2.4 ± 0.1	2.2 ± 0.5	2.3 ± 0.2	2.3 ± 0.1
	Race Jockey	2.3 ± 0.1	2.1 ± 0.6	2.3 ± 0.1	2.3 ± 0.1
	ES (Trials)	0.2	0.7	0.0	0.3
	ES (Jockey)	0.7	0.3	0.1	0.0
Displacement (m)	Trials Horse	0.102 ± 0.023^{+}	0.078 ± 0.023^{+}	0.149 ± 0.04^{+}	0.114 ± 0.028^{+}
	Trials Jockey	0.067 ± 0.027	0.023 ± 0.007	0.037 ± 0.016	0.054 ± 0.021
	Race Jockey	0.055 ± 0.023*	0.028 ± 0.010*	0.056 ± 0.025*	0.056 ± 0.022
	ES (Trials)	2.0	4.6	5.2	3.4
	ES (Jockey)	0.7	0.8	1.3	0.1
Mean linear acceleration ($m{\cdot}s^{-2}$)	Trials Horse	45.3 ± 14.6^{+}	33.1 ± 13.8^{+}	53.7 ± 17.6^{+}	37.5 ± 8.4^{+}
	Trials Jockey	22.2 ± 8.0	8.2 ± 2.4	10.6 ± 3.4	16.1 ± 5.6
	Race Jockey	21.4 ± 9.0	9.3 ± 2.9*	13.9 ± 5.3*	15.8 ± 5.2
	ES (Trials)	2.8	3.6	4.8	4.2
	ES (Jockey)	0.1	0.6	1.1	0.1
Peak linear acceleration ($m{\cdot}s^{-2}$)	Trials Horse	74.5 ± 16.6^{+}	65.0 ± 16.1^{+}	87.5 ± 19.1^{+}	68.9 ± 11.3^{+}
	Trials Jockey	33.8 ± 11.3	14.6 ± 5.6	16.9 ± 5.1	25.3 ± 8.4
	Race Jockey	36.7 ± 17.1	16.6 ± 6.0*	21.4 ± 7.4*	28.1 ± 12.6
	ES (Trials)	4.1	5.9	7.1	6.2
	ES (Jockey)	0.3	0.5	1.0	0.4
Mean time difference** (s)	Trials	-0.025 ± 0.061	-0.016 ± 0.059	-0.003 ± 0.064	-0.027 ± 0.051

** Difference in time between jockey and horse minimum displacement. A positive time difference was obtained when the jockey's movement followed the horse and a negative value when the jockey preceded horse movement. Means with differing superscripts differ ($p < 0.05$) between horse and jockey at trials ($^{+}$) and between jockey riding at trials and races (*).
