# Supplementary material for: Physiological Demands and Muscle Activity of Jockeys in Trial and Race Riding
Source: Animals (Basel). 2022 Sep 8;12(18):2351. doi: 10.3390/ani12182351 (PMC9495223; doi:10.3390/ani12182351)
Supplement: Supplementary file 1 [file animals-12-02351-s001.zip › Table S1.pdf]

Table S1. Horse contact rating scale and Borg's rating of perceived exertion 10-point scale used with jockeys (n = 10) to score each ride of a morning's track-work. * indicates midway between two categories.

Horse Contact	Scale	Borg RPE
Kick on	0	Nothing at all
Kick a bit	1	Very, very easy
*	2	Easy
No pull, no kick on	3	Moderate
*	4	Somewhat Hard
Pulls a bit	5	Hard
*	6	*
Keep a hold	7	Very Hard
*	8	*
Hold hard	9	*
Can't hold/stop	10	Maximal

Terminology:

Kick on	Horse needs encouragement to maintain its pace
No pull, No kick on	Horse is 'on the bridle' and travels at a consistent pace with a light rein contact and no encouragement
Pulls a bit	Horse requires rider to maintain a steady pressure on the reins otherwise it would increase speed
Keep a hold	Rider required to maintain a strong pressure bridge hold on the reins to prevent horse increasing speed
Hold hard	Rider required to maintain a maximum pressure bridge hold on the reins to prevent horse increasing speed
Can't hold/stop	Horse increases speed irrespective of rider
